# Universal health care no guarantee of equity: Comparison of socioeconomic inequalities in the receipt of coronary procedures in patients with acute myocardial infarction and angina

**DOI:** 10.1186/1471-2458-9-460

**Published:** 2009-12-14

**Authors:** Rosemary J Korda, Mark S Clements, Chris W Kelman

**Affiliations:** 1 Australian Centre for Economic Research on Health, The Australian National University, Canberra ACT 0200, Australia; 2 National Centre for Epidemiology and Population Health, The Australian National University, Canberra ACT 0200, Australia

## Abstract

**Background:**

In Australia there is a socioeconomic gradient in morbidity and mortality favouring socioeconomically advantaged people, much of which is accounted for by ischaemic heart disease. This study examines if Australia's universal health care system, with its mixed public/private funding and delivery model, may actually perpetuate this inequity. We do this by quantifying and comparing socioeconomic inequalities in the receipt of coronary procedures in patients with acute myocardial infarction (AMI) and patients with angina.

**Methods:**

Using linked hospital and mortality data, we followed patients admitted to Western Australian hospitals with a first admission for AMI (n = 5539) or angina (n = 7401) in 2001-2003. An outcome event was the receipt, within a year, of a coronary procedure—angiography, angioplasty and/or coronary artery bypass surgery (CABG). Socioeconomic status was assigned to each individual using an area-based measure, the SEIFA Index of Disadvantage. Multivariable proportional hazards regression was used to model the association between socioeconomic status and procedure rates, allowing for censoring and adjustment of multiple covariates. Mediating models examined the effect of private health insurance.

**Results:**

In the AMI patient cohort, socioeconomic gradients were not evident except that disadvantaged women were more likely than advantaged women to undergo CABG. In contrast, in the angina patient group there were clear socioeconomic gradients for all procedures, favouring more advantaged patients. Compared with patients in the most disadvantaged quintile of socioeconomic status, patients in the least disadvantaged quintile were 11% (1-21%) more likely to receive angiography, 52% (29-80%) more likely to undergo angioplasty and 30% (3-55%) more likely to undergo CABG. Private health insurance explained some of the socioeconomic variation in rates.

**Conclusions:**

Australia's universal health care system does not guarantee equity in the receipt of high technology health care for patients with ischaemic heart disease. While such a system might ensure equity for patients with AMI, where guidelines for treatment are relatively well established, this is not the case for angina patients, where health care may be less urgent and more discretionary.

## Background

In Australia, like elsewhere, there is a socioeconomic gradient in health, with mortality rates highest amongst socioeconomically disadvantaged individuals.[1] Much of this inequality is attributable to causes that are potentially avoidable through effective and timely health care,[2]. the leading cause being ischaemic heart disease (IHD).[3] However, a common view is that the health care system plays little role in generating these health inequalities, particularly in countries like Australia with universal health insurance systems.[4,5] Indeed, Australia's universal insurance scheme, Medicare, provides free treatment to patients treated in public hospitals and subsidises privately-delivered medical care, aiming 'to give all Australians, regardless of their personal circumstances, access to health care at an affordable cost or at no cost'.[6]

Nevertheless, as the WHO Commission on Social Determinants of Health reported recently, the opportunities for health systems to mitigate the harmful health effects of social stratification are all too often missed, and that in some instances they actually perpetuate inequity.[7] This may be true even in universal systems, particularly those where private insurance and direct private payments play some role in access to services.[8] Indeed, another underlying principle of Australia's universal system is to also allow '...choice for individuals through substantial private sector involvement in delivery and financing.'[6]. This 'choice' principle is somewhat at odds with the equity principle as it is essentially made available to those who can afford private health insurance (PHI), which covers some or all of the out-of-pocket cost of being treated as a private patient, in a public or private hospital. Around half of the population hold private insurance, 76% of households in the highest income quintile, and 23% in the lowest.[9] This, combined with non-system characteristics such as socioeconomic differentials in patient health literacy and expectations,[10,11] suggests that those who are more socioeconomically advantaged may derive more benefit from the system than those who are less advantaged. This may be particularly true for care where there is more choice involved.

This study examines socioeconomic inequalities in the receipt of high technology health care in patients with IHD in Australia, to examine the possibility that the health care system may actually perpetuate inequalities in IHD. Ischaemic heart disease is the leading cause of avoidable mortality in Australia[12] and the main contributor to the socioeconomic mortality gap.[3] Our main aim is to quantify socioeconomic inequalities in the receipt of coronary procedures, including angiography and revascularisation procedures—angioplasty and coronary artery bypass grafting (CABG)—which are known to improve survival in patients with IHD.[13] A second aim is to explore the hypothesis that inequalities will be greater where there is relatively more discretion around treatment and hence more scope for variation in provision of services.[14] We do this by examining inequalities separately in patients with acute myocardial infarction (AMI), a relatively well-defined clinical condition where the guidelines for intervention are now relatively well established, and in patients with angina, where this is less the case. We also explore inequalities in the angina patient group stratified by emergency and elective admission status as it is likely that emergency patients are largely presenting with unstable angina, where care is perhaps less discretionary compared with elective patients, who are more likely to have chronic stable angina. A third aim is to examine the extent to which private health care and private health insurance explain any inequality in receipt of procedures.

## Methods

We used administrative hospital and death data from Western Australia (WA). Western Australia comprises a tenth of the total Australian population.[15] In 2004 (the last year of data for the present study), the population was 1.98 million, three-quarters of whom resided in metropolitan Perth.[16] The health system in WA is typical of Australia, however revascularisation rates differ from other states: age-adjusted CABG and total revascularisation rates for WA in 2000 (unadjusted for need) were the lowest of any state or territory, while PTCA rates were the second highest.[17]

Data were extracted from theHospital Morbidity Data System, which contains information on each hospital admission in WA, including sociodemographic characteristics, clinical diagnoses and medical and surgical procedures. The dataset covers all public and private hospitals, including acute care hospitals and day surgeries. In addition, records were extracted from the Mortality Data System, which records all deaths registered in WA. An established population-based record linkage system allows linking of records relating to individual patients both within and between these datasets.

**Figure 1:**
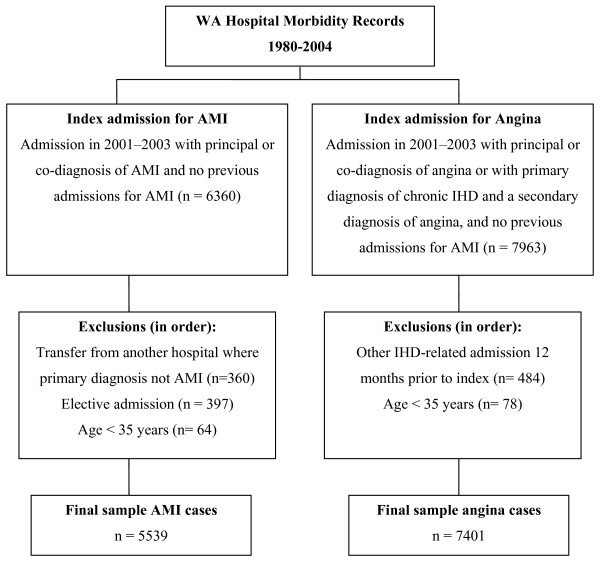
**Case selection**.

For this study, an AMI case was a patient with an AMI index admission, defined as an admission to hospital between 2001 and 2003 with a principal or co-diagnosis of AMI (ICD-10 code: I2I) and with no previous admissions for AMI recorded for that patient (linked records were available from 1980 onward). For angina cases, an index admission was defined as an admission to hospital between 2001 and 2003 with a principal or co-diagnosis of angina (ICD-10 I20 - 99% of cases; 'other acute IHD' (I24)); or a primary diagnosis of chronic IHD (I25) with a secondary diagnosis of angina (I20), and no previous admissions for AMI. To select the final sample of cases, further exclusions were applied, as shown in Figure 1. The selection criteria and codes used are adapted from established methods.[14] The final sample sizes were 5539 for AMI and 7401 for angina.

The eleven procedure code and procedure date fields were used to identify the outcome event, which was receipt of a coronary procedure—angiography (ICD-10 codes 38215, 38218), PTCA (ICD-10 codes 35304, 35305, 35310) and/or CABG (ICD-10 codes 38497, 38500, 38503, 90201)—within 12 months of the index admission. The main explanatory variable, socioeconomic status (SES), was assigned to each individual using the SEIFA Index of Disadvantage, a census-based measure of area-level SES.[18] The Index was assigned at the level of the Collector District (CD), the smallest census unit available, using the 2001 version of the SEIFA. Where the CD information was missing (17% of cases), the CD of the admission closest in time to the index admission was used (4% of cases), and where this was unavailable postcode was used. A SEIFA score could be assigned to 97% of cases. After assigning a score, patients were classified into population-based quintiles of SES.

Non-SES variables in the hospital dataset that may be associated with both SES and receipt of coronary procedures and hence that may confound the inequality estimates were included in the adjusted analyses. These were: *Age Group *(ten-year age groups from 35-44 to 75-84 then 85 plus), *Sex*, *Marital Status *(single or married/defacto), Aboriginal/Torres Strait Islander (*ATSI*) status (ATSI or non-ATSI), *Country of Birth *(Australia/New Zealand or other), *Area of Residence *(based on Accessibility/Remoteness Index of Australia (ARIA+) categories: major cities, inner regional and more remote), *Hospital Area *of index admission (metropolitan or rural), and *Comorbidity*. *Comorbidity *was measured using the modified Charlson Index, which is a weighted index based on diagnoses for all hospitalisations in the 12 months prior to the index admission.[19,20] It is included as a potential confounder because lower SES is associated with higher comorbidity, and those with comorbidity are less likely to be offered a procedure.[21]

Two variables—*Private Insurance *(those holding private hospital insurance or who had hospital cover with the Department of Veteran's Affairs versus none) and *Private Hospital *(private hospital admission versus public)—were treated as mediating variables, as per the third aim of the study, i.e. to examine the extent to which these variables explain any of the SES variation in receipt of procedures.

Admission-date and procedure-date variables were used to calculate time to procedure. Where procedure dates were missing (11.6%), the admission date for the admission in which the procedure was performed was substituted for the procedure date. The admission-type data element describes whether the admission occurred on an emergency basis (admission should occur within 24 hours) or an elective basis (either from a waiting list or not).[22] In this study, the variable was dichotomised as either emergency or elective and was used to select AMI cases (i.e. emergency admission only, see Figure 1) and to stratify angina cases into emergency and elective admissions.

### Analysis

Survival analysis methods were used to analyse the data, where survival is the time between the first day of the index admission and the date of a subsequent coronary procedure. Data were right-censored when the patient had a subsequent myocardial infarction, he/she died, or 12 months had elapsed since the index admission. For analysis of PTCA and CABG, censoring also occurred when the patient had the alternative procedure because once patients are offered PTCA they are no longer candidates for CABG, in the short-term at least, and vice-versa. Censoring was assumed to be uninformative.

To provide an initial summary of the data by sex and SES, the crude probability of receiving a procedure by 12 months was estimated using the Kaplan-Meier method. Cox proportional hazards regression was used to model the association between SES and procedure rates, allowing adjustment for age (Model 1), all confounders (Model 2) and confounding and mediating variables (Model 3). Hazard ratios (HRs) were calculated for each SES quintile, with the most disadvantaged quintile as the reference group. The proportional hazards assumption was tested for all models by calculating Schoenfeld residuals. Where covariates displayed non-proportionality of hazards, a stratified form of the model was used.

For the AMI patients, multivariate Cox models were run separately for males and females. This is because the distribution of procedures by SES differed across males and females, with generally linear SES trends for males and U-shaped distributions for females (linear for CABG). These differences were confirmed statistically by modelling male and females together and finding the sex by SES interaction terms to be jointly significant (F-test of joint interaction terms, p < .01 for all models except CABG). For the angina patients, there were socioeconomic gradients for all procedures in both male and female patients, with the more advantaged patients more likely to receive procedures. That the inequality patterns were similar across the sexes was confirmed statistically (when male and females were modelled together the sex by SES interaction terms were not jointly significant: p-values for F-test of joint interaction terms: angiography, p = .325; PTCA, p = .567; CABG, p = .157; and CARP, p = .976.). Thus, to increase power and simplify presentation of results, males and females were modelled together with sex entered as a covariate in these models. STATA 9.0 statistical software was used for all analyses.[23]

The project was approved by The Australian National University Human Research Ethics Committee.

## Results

Sample characteristics are shown in Table 1. The sample profile is similar across the AMI and angina groups with a few exceptions. The most notable are that the angina group were less likely to have comorbidity (26% versus 44%), more likely to have private insurance (45% compared to 35%) and more likely to be admitted to a private hospital for the index admission (38% compared to 20%).

**Table 1:** Sample characteristics for patients admitted to hospital with acute myocardial infarction (AMI) or angina, 2001-03

	AMI patients (n = 5539)		Angina patients (n = 7401)	
		
	n	%	n	%
**Patient**				
Sex				
Male	3637	65.7	4457	60.2
Female	1902	34.3	2944	39.8
Age Group^a^				
35-44	307	5.5	409	5.5
45-54	889	16.1	1278	17.3
55-64	1091	19.7	1815	24.5
65-54	1247	22.5	2039	27.6
75-84	1302	23.5	1455	19.7
85 plus	703	12.7	405	5.5
SES				
Q1	1322	24.5	1645	22.8
Q2	1227	22.8	1593	22.1
Q3	1028	19.1	1441	20.0
Q4	913	16.9	1248	17.3
Q5	901	16.7	1295	17.9
Country of Birth				
Aus/NZ	3068	57.7	4337	60.8
Not Aus/NZ	2247	42.3	2791	39.2
Aboriginal				
ATSI	189	3.4	209	2.8
Not ATSI	5350	96.6	7192	97.2
Area of Residence				
Major cities	3980	73.8	5055	70.0
Inner regional	636	11.8	1000	13.8
More remote	777	14.4	1170	16.2
Marital Status				
Single	1932	35.9	2217	30.5
Married/defacto	3444	64.1	5046	69.5
Comorbidity				
0	3113	56.2	5459	73.8
1	1324	23.9	1227	16.6
2	490	8.9	369	5.0
3 or more	612	11.1	346	4.7
Private Insurance				
Yes	1920	34.7	3363	45.4
No	3619	65.3	4038	54.6
**Hospital**				
Hospital Area				
Metropolitan	4504	81.3	6332	85.6
Rural	1035	18.7	1069	14.4
Private hospital				
No, Public	4440	80.2	4599	62.1
Yes, Private	1099	19.8	2802	37.9

^a ^Mean age of AMI patients = 67.6 (standard deviation 14.2, range 35-104) (Males: 64.3, SD: 13.6; Females: 73.8, SD: 13.1). Mean age of angina patients = 65.0 (standard deviation 12.46, range 35-103) (Males: 63.0, SD: 12.0; Females: 68.0, SD: 12.8).

Notes.

1. ATSI = Aboriginal or Torres Strait Islander

2. Comorbidity refers to modified Charlson Index score.

3. Missing data in AMI patients: SES, n = 148 (2.7%); country of birth, n = 224 (4.0%); area of residence, n = 146 (2.7%); marital status, n = 163 (2.9%); total no. of patients with missing data on at least one variable = 479 (8.7%; 9.2% in males, 7.7% in females). Missing data in angina patients: SES, n = 179 (2.4%); country of birth, n = 273 (3.7%); area of residence, n = 176 (2.4%); marital status, n = 138 (1.9%); total no. of patients with missing data on at least one variable = 554 (7.5%).

4. Among AMI patients, 99% of those living in major cities, 54% of inner regional patients and 20% of more remote patients were admitted to a metropolitan hospital for the index admission, with corresponding figures for angina patients being 99%, 75% and 41%. All coronary procedures were performed in metropolitan hospitals, with most performed in public hospitals--86% of those in AMI patients and 56% of those in angina patients. Over 90% of AMI patients first admitted to a public hospital also had their procedure in a public hospital, while of those first admitted to a private hospital, 33% had their angiography, 27% their PTCA and 49% their CABG in a public hospital.

### Coronary procedures in AMI patients

The probabilities of having a procedure by socioeconomic quintile and sex for AMI patients are shown in Table 2 (males) and Table 3 (females). The overall probability of a male receiving an angiogram within one year of the index admission was 76% and of receiving any coronary artery revascularisation procedure (CARP) was 56%—46% for PTCA and 19% for CABG. Median times to procedure were 3, 3 and 19 days for angiography, PTCA and CABG, respectively (where day 1 is the day of admission), with 90% of procedures carried out within 42, 35 and 166 days, respectively. For females, the probability of angiography was 50%, while for CARP it was 30%—25% for PTCA and 8% for CABG. Median times to procedure were 3, 3 and 29 days for angiography, PTCA and CABG, respectively, with 90% of procedures carried out within 43, 33 and 213 days, respectively. Female procedure rates were significantly lower than male rates for all procedures after adjusting for age (p < .001 for all procedures).

**Table 2:** Probability of procedures (%) by socioeconomic quintile (Q) and hazard ratios for male patients with acute myocardial infarction

		**Model 1**			**Model 2**			**Model 3**		
				
	**%**	**HR**	**95% CI**	**p**	**HR**	**95% CI**	**p**	**HR**	**95% CI**	**p**
				
**Angiography**										
SES Q1	76.7	1.00	-	-	1.00	-	-	1.00	-	-
SES Q2	75.5	1.05	0.94-1.17	.423	1.01	0.90-1.14	.846	1.02	0.90-1.15	.744
SES Q3	75.1	1.01	0.90-1.14	.895	0.98	0.86-1.11	.699	0.98	0.86-1.11	.739
SES Q4	75.0	1.05	0.93-1.18	.448	0.94	0.82-1.07	.359	0.95	0.83-1.09	.456
SES Q5	76.3	1.14	1.01-1.29	.041	0.95	0.83-1.09	.507	0.99	0.86-1.14	.886
**Total**	75.6		**Trend**	.080		**Trend**	.281		**Trend**	.542
**PTCA**										
SES Q1	43.3	1.00	-	-	1.00	-	-	1.00	-	-
SES Q2	46.6	1.15	0.99-1.33	.072	1.09	0.93-1.27	.281	1.11	0.95-1.29	.204
SES Q3	44.2	1.08	0.93-1.27	.317	1.05	0.89-1.24	.579	1.03	0.88-1.22	.690
SES Q4	49.9	1.27	1.08-1.49	.003	1.11	0.94-1.32	.207	1.13	0.96-1.34	.150
SES Q5	48.4	1.30	1.10-1.52	.002	1.01	0.85-1.21	.879	1.04	0.87-1.24	.688
**Total**	46.1		**Trend**	.001		**Trend**	.755		**Trend**	.594
**CABG**										
SES Q1	21.0	1.00	-	-	1.00	-	-	1.00	-	-
SES Q2	18.5	0.95	0.71-1.26	.720	0.93	0.69-1.27	.660	0.93	0.69-1.26	.633
SES Q3	20.9	1.03	0.77-1.37	.851	0.98	0.72-1.34	.922	0.96	0.70-1.31	.788
SES Q4	17.0	0.85	0.61-1.18	.325	0.86	0.61-1.21	.374	0.82	0.58-1.16	.261
SES Q5	17.2	0.97	0.70-1.35	.872	1.03	0.73-1.47	.850	0.97	0.67-1.39	.854
**Total**	19.0		**Trend**	.655		**Trend**	.874		**Trend**	.572
**CARP**										
SES Q1	54.7	1.00	-	-	1.00	-	-	1.00	-	-
SES Q2	55.7	1.10	0.96-1.25	.171	1.06	0.92-1.22	.392	1.07	0.93-1.23	.336
SES Q3	55.3	1.07	0.93-1.24	.311	1.04	0.90-1.21	.562	1.03	0.89-1.19	.711
SES Q4	57.7	1.17	1.02-1.35	.030	1.06	0.91-1.24	.422	1.06	0.91-1.24	.425
SES Q5	56.6	1.22	1.06-1.41	.006	1.03	0.88-1.21	.688	1.03	0.88-1.21	.679
**Total**	55.7		**Trend**	.004		**Trend**	.672		**Trend**	.700

Notes.

1. PTCA = percutaneous transluminal coronary angioplasty; CABG = coronary artery bypass grafting; CARP = coronary artery revascularisation procedure (PTCA or CABG).

2. SES Q1 is most disadvantaged quintile.

3. SES Q1, n = 853; SES Q2, n = 810; SES Q3, n = 685; SES Q4, n = 586; SES Q5, n = 597.

4. Cumulative probability of a procedure at one year was estimated using the Kaplan-Meier estimator.

5. Because some patients had both CABG and PTCA on the same day, the % of patients undergoing first CARP procedures does not equal % PTCA plus % CABG.

6. Hazard ratios estimated using Cox regression and adjusted for age in Model 1, as well as other confounding variables (country of birth, Aboriginal/Torres Strait Islander status, marital status, comorbidities, area of residence and hospital area) in Model 2 and confounding and mediating variables (private insurance and private hospital) in Model 3.

**Table 3:** Probability of procedures (%) by socioeconomic quintile (Q) and hazard ratios for female patients with acute myocardial infarction

		**Model 1**			**Model 2**			**Model 3**		
				
	**%**	**HR**	**95% CI**	**p**	**HR**	**95% CI**	**p**	**HR**	**95% CI**	**p**
				
**Angiography**										
SES Q1	50.1	1.00	-	-	1.00	-	-	1.00	-	-
SES Q2	54.0	1.14	0.94-1.38	.172	1.17	0.96-1.43	.127	1.15	0.94-1.41	.162
SES Q3	56.6	1.37	1.12-1.67	.002	1.37	1.11-1.69	.003	1.31	1.06-1.62	.011
SES Q4	50.4	1.12	0.91-1.39	.278	1.05	0.84-1.31	.686	1.04	0.83-1.31	.731
SES Q5	36.8	1.05	0.82-1.33	.700	0.91	0.71-1.17	.469	0.87	0.67-1.12	.274
**Total**	50.1		**Trend**	.375		**Trend**	.675		**Trend**	.463
**PTCA**										
SES Q1	21.5	1.00	-	-	1.00	-	-	1.00	-	-
SES Q2	30.0	1.43	1.09-1.88	.011	1.39	1.04-1.85	.026	1.38	1.03-1.84	.030
SES Q3	28.7	1.47	1.10-1.96	.009	1.53	1.13-2.07	.006	1.50	1.11-2.04	.009
SES Q4	27.4	1.39	1.03-1.88	.030	1.30	0.95-1.79	.103	1.37	0.99-1.89	.057
SES Q5	18.3	1.20	0.85-1.70	.291	1.08	0.75-1.55	.680	1.03	0.71-1.49	.873
**Total**	25.3		**Trend**	.151		**Trend**	.456		**Trend**	.495
**CABG**										
SES Q1	11.4	1.00	-	-	1.00	-	-	1.00	-	-
SES Q2	9.1	0.73	0.44-1.23	.240	0.83	0.49-1.42	.497	0.83	0.49-1.42	.504
SES Q3	9.7	0.83	0.49-1.41	.485	0.76	0.43-1.35	.345	0.76	0.43-1.35	.345
SES Q4	5.8	0.54	0.29-1.02	.057	0.49	0.24-0.97	.041	0.49	0.24-1.00	.051
SES Q5	4.3	0.52	0.25-1.09	.085	0.43	0.19-0.95	.037	0.43	0.19-0.97	.042
**Total**	8.4		**Trend**	.034		**Trend**	.009		**Trend**	.013
**CARP**										
SES Q1	29.6	1.00	-	-	1.00	-	-	1.00	-	-
SES Q2	35.6	1.24	0.97-1.58	.080	1.23	0.96-1.59	.107	1.22	0.95-1.58	.121
SES Q3	34.7	1.29	1.00-1.67	.048	1.30	1.00-1.71	.052	1.27	0.97-1.70	.081
SES Q4	31.4	1.17	0.90-1.53	.248	1.11	0.83-1.47	.484	1.17	0.88-1.57	.283
SES Q5	21.1	1.00	0.73-1.37	.989	0.89	0.64-1.24	.506	0.85	0.85-1.19	.346
**Total**	30.1		**Trend**	.727		**Trend**	.716		**Trend**	.687

Notes.

1. PTCA = percutaneous transluminal coronary angioplasty; CABG = coronary artery bypass grafting; CARP = coronary artery revascularisation procedure (PTCA or CABG).

2. SES Q1 is most disadvantaged quintile.

3. SES Q1, n = 469; SES Q2, n = 417; SES Q3, n = 343; SES Q4, n = 327; SES Q5, n = 304.

4. Cumulative probability of a procedure at one year was estimated using the Kaplan-Meier estimator.

5. Because some patients had both CABG and PTCA on the same day, the % of patients undergoing first CARP procedures does not equal % PTCA plus % CABG.

6. Hazard ratios estimated using Cox regression and adjusted for age in Model 1, as well as other confounding variables (country of birth, Aboriginal/Torres Strait Islander status, marital status, comorbidities, area of residence and hospital area) in Model 2 and confounding and mediating variables (private insurance and private hospital) in Model 3.

Adjusting for age alone (Model 1, Table 2), angiography rates for males were slightly higher in Q5 than Q1 (HR = 1.14; 95% CI: 1.01-1.29) but the overall test for SES trend was not significant (p = .080). For PTCA (and CARP), but not CABG, there was a socioeconomic gradient in age-adjusted procedure rates favouring high SES patients (test for trend, p = .001, .004 and .655, for PTCA, CARP and CABG, respectively). However, after simultaneously adjusting for all of the confounding factors (Model 2, Table 2) there were no significant associations between SES and receipt of any of the coronary procedures. After entering the mediating variables—*Private Insurance *and *Private Hospital*—into the fully-adjusted models (Model 3, Table 2) there was virtually no change in the SES estimates for any of the procedures.

For females, those in the middle quintiles were the most likely to receive angiography and PTCA (or CARP), after adjusting for age alone (Model 1, Table 3) and multiple confounders (Model 2, Table 3). For CABG there was a significant socioeconomic gradient, with advantaged women less likely to receive this procedure than more disadvantaged women, even after adjustment for confounding (test for SES trend p = .009). As with the male patient group, when *Private Insurance *and *Private Hospital *were also entered into the model (Model 3, Table 3) there was virtually no change in the SES estimates for any of the procedures.

### Coronary procedures in angina patients

The probability of a male patient presenting with angina receiving an angiogram within one year of the index admission was 77% and of receiving any revascularisation procedure (CARP) was 45%—28% for PTCA and 24% for CABG. Median times to procedure were 1 day (day of admission), 5 and 23 days for angiography, PTCA and CABG, respectively, with 90% of procedures carried out within 11, 43 and 112 days, respectively. For female patients with angina, the probability of angiography was 63%, while for CARP it was 24%—17% for PTCA and 10% for CABG. Median times to procedure were 1, 6 and 26 days for angiography, PTCA and CABG, respectively, with 90% of procedures carried out within 24, 65 and 119 days, respectively. Female procedure rates were consistently lower than male rates and remained so after adjusting for age (p < .001 for all procedures).

The probabilities of having a procedure by socioeconomic quintile, for males and females combined, are shown in Table 4. The probability of angiography was 71%, while for CARP it was 36%—23% for PTCA and 18% for CABG. After adjusting for age alone (Model 1, Table 4), the SES tests for trend were significant for all procedures, with procedure rates increasing with increasing SES. After adjusting for all confounding variables (Model 2, Table 4), patients in Q5 were 11% (95% CI: 1-21%) more likely to receive angiography and 41% (95% CI: 24-61%) more likely to receive a revascularisation procedure than those in Q1—they were 52% (95% CI: 29-80%) more likely to receive PTCA, and 30% (95% CI: 3-55%) more likely to undergo CABG.

**Table 4:** Probability of procedures (%) by socioeconomic quintile (Q) and hazard ratios for patients with angina

		**Model 1**			**Model 2**			**Model 3**		
				
	**%**	**HR**	**95% CI**	**p**	**HR**	**95% CI**	**p**	**HR**	**95% CI**	**p**
				
**Angiography**										
SES Q1	67.7	1.00	-	-	1.00	-	-	1.00	-	-
SES Q2	68.3	1.04	0.96-1.13	.361	1.02	0.94-1.11	.667	1.03	0.94-1.12	.550
SES Q3	72.3	1.12	1.03-1.22	.008	1.05	0.96-1.14	.305	1.05	0.96-1.15	.287
SES Q4	74.0	1.21	1.11-1.32	<.001	1.08	0.98-1.18	.118	1.08	0.98-1.18	.118
SES Q5	75.0	1.27	1.16-1.38	<.001	1.11	1.01-1.21	.033	1.08	0.98-1.19	.131
**Total**	70.6		**Trend**	<.001		**Trend**	.017		**Trend**	.074
**PTCA**										
SES Q1	18.2	1.00	-	-	1.00	-	-	1.00	-	-
SES Q2	22.3	1.23	1.05-1.44	.009	1.24	1.05-1.46	.009	1.20	1.02-1.42	.029
SES Q3	24.7	1.39	1.18-1.62	<.001	1.39	1.18-1.63	<.001	1.31	1.11-1.54	.002
SES Q4	25.2	1.43	1.21-1.68	<.001	1.34	1.13-1.58	.001	1.23	1.03-1.47	.019
SES Q5	28.3	1.65	1.41-1.93	<.001	1.52	1.29-1.80	<.001	1.32	1.10-1.57	.003
**Total**	23.4		**Trend**	<.001		**Trend**	<.001		**Trend**	.005
**CABG**										
SES Q1	15.3	1.00	-	-	1.00	-	-	1.00	-	-
SES Q2	17.8	1.16	0.96-1.40	.115	1.12	0.93-1.36	.228	1.11	0.92-1.35	.277
SES Q3	17.3	1.12	0.92-1.35	.259	1.07	0.88-1.30	.493	1.04	0.86-1.28	.654
SES Q4	20.4	1.36	1.13-1.65	.001	1.28	1.05-1.56	.014	1.20	0.99-1.47	.069
SES Q5	20.5	1.38	1.14-1.66	.001	1.30	1.03-1.55	.024	1.14	0.93-1.42	.211
**Total**	17.7		**Trend**	<.001		**Trend**	.010			.144
**CARP**										
SES Q1	30.2	1.00	-	-	1.00	-	-	1.00	-	-
SES Q2	35.5	1.20	1.06-1.35	.003	1.18	1.05-1.34	.008	1.15	1.01-1.31	.025
SES Q3	37.2	1.27	1.12-1.43	<.001	1.25	1.10-1.41	.001	1.19	1.05-1.35	.007
SES Q4	39.9	1.40	1.24-1.59	<.001	1.30	1.15-1.49	<.001	1.20	1.06-1.38	.006
SES Q5	42.4	1.53	1.36-1.73	<.001	1.41	1.24-1.61	<.001	1.22	1.07-1.90	.004
**Total**	36.4		**Trend**	<.001		**Trend**	<.001		**Trend**	.004

Notes.

1. PTCA = percutaneous transluminal coronary angioplasty; CABG = coronary artery bypass grafting; CARP = coronary artery revascularisation procedure (PTCA or CABG).

2. SES Q1 is most disadvantaged quintile.

3. SES Q1, n = 1645; SES Q2, n = 1593; SES Q3, n = 1441; SES Q4, n = 1248; SES Q5, n = 1295.

4. Cumulative probability of a procedure at one year was estimated using the Kaplan-Meier estimator.

5. Because some patients had both CABG and PTCA on the same day, the % of patients undergoing first CARP procedures does not equal % PTCA plus % CABG.

6. Hazard ratios estimated using Cox regression and adjusted for age in Model 1, as well as other confounding variables (country of birth, Aboriginal/Torres Strait Islander status, marital status, comorbidities, area of residence and hospital area) in Model 2 and confounding and mediating variables (private insurance and private hospital) in Model 3.

7. Proportional hazards assumption violated for CABG (p-value for global test of proportional hazards assumption = .006) in Model 2.

The extent to which private health care might explain the SES inequality in procedure rates was examined. Crude associations between SES and *Private Insurance *and *Private Hospital *showed significant linear trends, with higher SES individuals more likely to hold insurance and be admitted to a private hospital. Having private insurance and being admitted to a private hospital were both associated with an increased likelihood of having a procedure. When these private care mediating variables were entered into the fully adjusted Cox proportional hazards model (Model 3, Table 4), the HRs for SES were all reduced, particularly in SES quintile 5 for PTCA and CABG. However, on formal testing none of the comparisons of SES estimates across the models with and without these mediators reached significance at the .05 level.

We also examined the extent to which angiography may have been the rate-limiting step in revascularisation, by including only those patients who had had angiography. This resulted in a slight reduction in inequality estimates, but significant inequalities remained, particularly notable for PTCA.

Finally, in a sub-analysis we explored inequalities in the angina patient group stratified by emergency (n = 4072) and elective (n = 3329) admission status. Compared with emergency patients, the elective patients had a relatively higher proportion of high SES patients (21.2% versus 15.2% in Q5), holders of private insurance (59.5% versus 34.0%) and private hospital admissions (54.7% versus 24.1%). Approximately half of the elective group (43.2%) were admitted from a waiting list. The probabilities of having a procedure by socioeconomic quintile, along with adjusted HRs (Model 2), are shown in Table 5. Procedure rates were higher in the elective than the emergency group. The pattern of inequality in the emergency patients is similar to the total angina group, with angiography and PTCA rates increasing with increasing SES, although receipt of CABG was not significantly associated with SES in these patients. In the elective patients, linear tests for SES trend were significant for all procedures. In contrast to the emergency admissions group, however, higher SES patients were less likely than lower SES patients to receive an angiogram, yet they were more likely to receive a revascularisation procedure.

**Table 5:** Probability of procedures (%) by socioeconomic quintile (Q) and adjusted hazard ratios for patients with angina, stratified by emergency and elective admission

	**Emergency admissions** **(n = 4072)**				**Elective admissions** **(n = 3329)**			
		
	**%**	**HR**	**95% CI**	**p**	**%**	**HR**	**95% CI**	**p**
		
**Angiography**								
SES Q1	50.3	1.00	-	-	95.3	1.00	-	-
SES Q2	50.2	1.03	0.90-1.17	.716	91.1	0.93	0.83-1.05	.249
SES Q3	56.1	1.15	1.00-1.31	.045	92.8	0.94	0.83-1.05	.271
SES Q4	56.6	1.14	0.99-1.31	.068	91.6	0.93	0.83-1.05	.233
SES Q5	58.2	1.21	1.05-1.40	.010	89.4	0.87	0.77-0.98	.021
**Total**	53.3		**Trend**	003	91.8		**Trend**	.037
**PTCA**								
SES Q1	14.7	1.00	-	-	24.0	1.00	-	-
SES Q2	17.4	1.20	0.94 -1.52	.140	28.8	1.27	1.01-1.59	.038
SES Q3	20.7	1.40	1.11-1.77	.005	30.0	1.34	1.06-1.67	.012
SES Q4	16.8	1.05	0.81-1.37	.715	34.2	1.54	1.23-1.92	<.001
SES Q5	23.1	1.49	1.16-1.92	.002	33.2	1.47	1.17-1.84	.001
**Total**	18.1		**Trend**	015	30.2		**Trend**	<.001
**CABG**								
SES Q1	9.3	1.00	-	-	25.5	1.00	-	-
SES Q2	10.8	1.17	0.85-1.62	.321	27.4	1.04	0.81-1.32	.776
SES Q3	9.4	1.01	0.72-1.41	.962	28.2	1.09	0.85-1.39	.491
SES Q4	11.1	1.28	0.90-1.81	.164	31.3	1.24	0.97-1.58	.085
SES Q5	9.7	1.04	0.71-1.53	.841	30.4	1.20	0.94-1.54	.139
**Total**	9.8		**Trend**	608	28.6		**Trend**	.049
**CARP**								
SES Q1	22.0	1.00	-	-	43.1	1.00	-	-
SES Q2	26.0	1.20	0.99-1.46	.059	47.4	1.15	0.98-1.36	.095
SES Q3	27.9	1.28	1.05-1.55	.013	48.9	1.20	1.03-1.42	.032
SES Q4	25.6	1.13	0.91-1.39	.271	54.2	1.37	1.16-1.62	<.001
SES Q5	29.9	1.35	1.09-1.67	.005	53.0	1.34	1.13-1.58	.001
**Total**	25.7		**Trend**	.020	49.5		**Trend**	<.001

Notes.

1. PTCA = percutaneous transluminal coronary angioplasty; CABG = coronary artery bypass grafting; CARP = coronary artery revascularisation procedure (PTCA or CABG).

2. SES Q1 is most disadvantaged quintile.

3. Emergency patients: SES Q1, n = 1008; SES Q2, n = 886; SES Q3, n = 805; SES Q4, n = 627; SES Q5, n = 596; Elective patients: SES Q1, n = 637; SES Q2, n = 707; SES Q3, n = 636; SES Q4, n = 621; SES Q5, n = 699.

4. Cumulative probability of a procedure at one year was estimated using the Kaplan-Meier estimator.

5. Because some patients had both CABG and PTCA on the same day, the % of patients undergoing first CARP procedures does not equal % PTCA plus % CABG.

6. Hazard ratios estimated using Cox regression and adjusted for age, country of birth, Aboriginal/Torres Strait Islander status, marital status, comorbidities, area of residence and hospital area (Model 2).

7. Proportional hazards assumption violated for CABG in elective admissions model (p-value for global test of proportional hazards assumption = .028).

## Discussion

The probability of receiving a coronary procedure in Western Australia varies by SES, with clear inequalities evident in patients admitted to hospital with angina but not in patients admitted for emergency AMI care. There were two exceptions in the findings, where socioeconomic gradients were reversed: in female patients admitted with AMI, more advantaged women were less likely to receive CABG than less advantaged women, and amongst elective angina patients, the advantaged women were less likely to have an angiogram. This may reflect true differences in need for the procedures, i.e., disadvantaged women may present with more severe disease. However, this cannot be ascertained from the study.

A strength of this study was the use of linked administrative data, which enabled individuals to be followed through time and data to be censored. Nevertheless there are limitations in using these data, which may have biased the results. First, several factors could have lead to either an underestimate or an overestimate of inequality. One is that while administrative data are highly reliable for ascertainment of coronary procedures, and the coding of AMI has been found to be reasonably reliable, this is not the case for angina—specificity and sensitivity is high but positive predictive value is relatively low.[24,25] Whether this would bias the inequality estimates depends on whether such misclassification is differential with respect to SES, which is unknown. Another possible bias is that while we adjusted for 'need' by limiting the study population to only those patients admitted with AMI or angina, appropriateness of care is complex and it is not possible to capture this complexity. The extent and direction of the potential bias this creates is difficult to predict as the relationship between procedure rates and disease and other characteristics is not straightforward, particularly for angina.[26]

Second, comorbidity may not have been fully accounted for in the models as there is considerable under-reporting of comorbidities in hospital admissions data.[27,28] This could have lead to an overestimation of inequality because lower SES patients are more likely to have comorbidities, and those with comorbidities are less likely to be offered a procedure.[21]

Third, several influences may have lead to an underestimation of inequality, including the use of area-level SES measures (rather than individual-level measures, which were not available), and the fact that only patients admitted to hospital were included—while this means all coronary procedures are captured, not all people with IHD who could potentially benefit from a coronary procedure are. This selection bias is less of a problem for AMI than for angina as most people who initially survive a heart attack present to hospital.[14] However, it is plausible that amongst those with angina, socioeconomically disadvantaged individuals are less likely to be admitted for investigation than the more advantaged. This is consistent with the study data that showed a relatively higher proportion of higher SES patients in the angina sample compared with the AMI sample.

There are few other studies with which the SES inequalities estimated for angina patients in this study can be directly compared. No previous Australian studies, and few international ones, have examined procedure rates in angina patients. Those that have, [29-31] like the current study, found evidence of inequalities. However, unlike the current study, the one study that compared inequalities in procedure rates across AMI and angina patients (Finland, 1995-98), found they were similar across the two groups.[30]

With regard to AMI patients, earlier Australian studies found overall coronary procedure rates to be higher in private than public hospitals (Victoria, 1995-1997)[32] and that socioeconomically advantaged patients were more likely to undergo angioplasty, but not CABG, than disadvantaged patients (Queensland, 1998).[33] Inequalities in procedure rates were also found in a study of patients with IHD followed up in a clinical trial of lipid-lowering medication (1990-1997).[34] Findings have been similar in international studies, though some studies have found no inequalities in procedure rates.[35-37] Most report inequalities in catheterised procedures (angiography and PTCA) and total revascularisation procedures, but not necessarily CABG. [29,30,38-42] Notably, at the time these other studies were carried out, percutaneous procedure rates were not used widely in patients with AMI. For example, in the earlier Australian studies the probability of angioplasty was less than 10%, compared with nearly 50% in this study. Limitations in directly comparing the earlier and the current studies notwithstanding, the difference in findings are not inconsistent with the inverse equity hypothesis, which predicts that inequalities will appear when there is still a relatively low rate of use in the population (as in the earlier studies), but will decrease as the intervention becomes more commonly used (as in the current study).[43]

That there was no clear evidence of socioeconomic inequality in coronary procedure rates in patients seeking emergency care following AMI should not be surprising. In Australia there are now relatively clear guidelines for the use of these procedures in this patient population, utilisation is relatively high, and there is free access to public hospital care—an environment that should present few financial barriers to receiving care. In the same context, that inequality exists in the receipt of coronary procedures in patients presenting with angina is perhaps not unexpected. The use of procedures in this population is more discretionary, a large proportion of patients are admitted electively, and a relatively large proportion of procedures are performed in private hospitals.

One of the possible mechanisms underlying socioeconomic inequalities amongst the angina patients—PHI—was explored in this study. As expected, higher SES patients were more likely to hold PHI and this increased the likelihood of receiving a procedure, although PHI did not fully account for the inequality in procedure rates. Notably, inequality was also evident in waiting times. Among the elective angina patients, lower SES patients were more likely to be admitted from a waiting list than higher SES patients (the percentages of patients admitted from a waiting list for Q1 (low SES) to Q5 (high SES), respectively, were: 64%, 51%, 44%, 36%, and 23%). This in turn was related to patients' PHI status, with nearly all (94%) patients without this insurance having to wait for the procedure, while the opposite was true for patients with private insurance (9% having to wait).

Patient and doctor characteristics, not examined in this study, may also explain the inequalities in procedure rates. First, there may be contraindications for receiving a procedure that are more prevalent in lower SES patients, but that were unmeasured in this study, including smoking,[44] obesity [44-46] and late presentation to hospital.[47,48] Second, disadvantaged patients may be less likely to see a specialist,[49] and specialists may be more likely than non-specialist doctors to recommend a coronary procedure.[50] Third, patients' preferences to seek care and undergo procedures may vary by SES—disadvantaged patients may have lower expectations[51] and be less willing to undergo a procedure[52] than more advantaged patients; and doctor's decisions may vary, either intentionally or unintentionally, depending on the social class of the patient, with higher SES patients at an advantage in this regard.[44,51,53]

That inequalities appeared for more discretionary care raises the question whether or not the higher procedure rates in advantaged individuals represent overuse, or whether they represent underuse in disadvantaged individuals. While these two possibilities have different implications for health inequalities, either state can be considered inequitable. In the case of underuse, disadvantaged individuals are not receiving health care from which they could benefit. In the case of over-use, this poses an overall problem for equity in a system with limited resources: where increases in health spending are increasingly going to more discretionary care, this leaves those with a greater capacity to benefit without, or having to wait longer for, much needed care, while the relatively 'well off' perhaps make more marginal gains.

## Conclusions

The findings suggest that universal health care systems such as Australia's, with mixed public/private funding and delivery allowing for 'choice' in heath care, may actually perpetuate health inequity. While such systems might ensure equity for patients with AMI, where guidelines for treatment are relatively well established, this is not the case for care of patients with angina, where high technology health care may be less urgent and more discretionary.

## Competing interests

The authors declare that they have no competing interests.

## Authors' contributions

RK conceived of the study, participated in the design of the study, performed the statistical analysis and drafted the manuscript. MC participated in the design of the study, advised on the statistical analysis and interpretation of results, and helped to draft the manuscript. CK participated in the design of the study and helped to draft the manuscript. All authors read and approved the final manuscript.

## Pre-publication history

The pre-publication history for this paper can be accessed here:

http://www.biomedcentral.com/1471-2458/9/460/prepub
